# Investigation and health risk assessment of PM_2.5_ during potato and meat frying

**DOI:** 10.1016/j.mex.2025.103419

**Published:** 2025-06-04

**Authors:** Mehdi Qasemi, Ali Alami, Ahmad Zarei, Mahmoud Shams, Mojtaba Afsharnia

**Affiliations:** a Environment Health Engineering, Student Workgroup of Social Development and Health Promotion Research Center, Gonabad University of Medical Sciences, Gonabad, Iran; b School of Medicine, Social Medicine Department, Social Determinants of Health Research Center, Gonabad University of Medical Sciences, Gonabad, Iran; c Department of Environmental Health Engineering, School of Public Health, Infectious Diseases Research Center, Gonabad University of Medical Sciences, Gonabad, Iran; d Social Determinants of Health Research Center, Mashhad University of Medical Sciences, Mashhad, Iran

**Keywords:** Food cooking, Indoor air quality, Oils, Particulate matters, Non-cancer risk, PAHs

## Abstract

Particulate matters (PM) are pollutants closely associated with human health in indoor and outdoor spaces. The present work was carried out to measure and estimate $PM_{2.5}$ levels and their relationship with human health through assessing non-carcinogenic risk during potato and meat frying. Levels of $PM_{2.5}$ during potato frying (at 150–240 °C) using sunflower oil and frying oil were in the ranges of 20–210 (mean 50.28), 20–180 (mean 46.94)$\;\mu g/m^{3}$, respectively. For meat frying, levels of $PM_{2.5}$ using sunflower oil and frying oil were in the ranges of 20–320 (mean 73.61) and 20–280 (mean 68.9)$\;\mu g/m^{3}$, respectively. The non-cancer risk due to the exposure to $PM_{2.5}$ using sunflower oil and frying oil during potato frying was in the ranges of 0.49–5.15 (mean 1.23) and 0.49–4.41 (mean 1.15) , respectively. For meat frying, the values of Hazard Quotients (HQ) using sunflower oil and frying oil were in the ranges of 0.49-7.84 (mean 1.80) and 0.49–6.86 (mean 1.69), respectively. Based on the mean values, non-cancer risk exceeded the acceptable limit of 1 for cooks, indicating risk to cooks from the exposure of PM during these cooking activities.

## Graphical abstract

**Figure:**
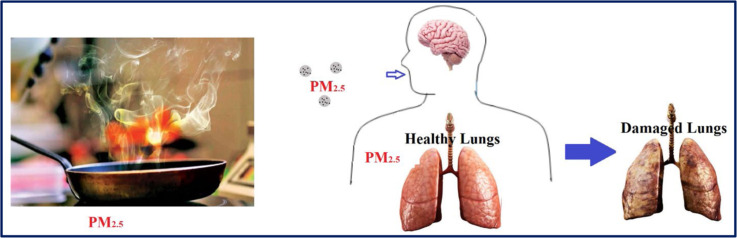

Specifications table

**Table:** 

Subject area:	Environmental Science
More specific subject area:	Indoor air quality
Name of your protocol:	$PM_{2.5}\;$during potato and meat frying
Reagents/tools:	Not applicable
Experimental design:	Particulate Air Monitoring Equipment, HAZ-DUST Model EPAM-5000, USA
Trial registration:	Not applicable
Ethics:	Not applicable
Value of the Protocol:	• Levels of $PM_{2.5}$ during potato and meat frying are investigated. • Levels of $PM_{2.5}$ during frying using two common cooking oils are investigated. • Non-cancer health risk was estimated. • Higher levels of $PM_{2.5}$ were found during meat frying at higher temperatures.

## Background

Outdoor and indoor air pollution are major causes of morbidity and mortality, causing many respiratory problems and other illnesses [[1], [2], [3]]. Food preparation and cooking activities may release many contaminants into foods and air [[4], [5], [6], [7]]. Among different types of contaminants, Particulate Matter (PM) is of great concern [8]. Particulate Matter ($PM_{2.5}$) is a term for liquid droplets and minute particles, equal to or less than 2.5 µm, in the air released from different sources both human activities or from natural processes [[9], [10], [11]]. $PM_{2.5}$ while inhaled through the mouth and nose can be accumulated in the respiratory tract and lungs, causing multiple health problems including eye irritation, skin allergies, and even lung cancer in long-term exposure [2,12]. It is reported that $PM_{2.5}$ has caused the greatest proportion of adverse health effects related to air pollution, both in the United States and globally [13]. Therefore in this study, we used the United States Environmental Protection Agency (US EPA) methodology to estimate the possible non-cancer risk to individuals caused by the inhalation of $PM_{2.5}$. The valuable results from the present research can be used as the basis by health professionals and policy makers in order to reduce indoor PM exposure and protect cooks to PM in similar settings worldwide.

## Description of protocol

### Assay procedure and quantitative analysis

In the present work to minimize experimental error, simulations in a real cooking conditions were considered, in order to study the release of $PM_{2.5}$ during potato and meat frying. Herein, the three potential influencing factors, including frying method, cooking temperatures (150, 190, and 240 °C), and two type of common cooking oils available in Iran’s supermarkets (sunflower oil as non-frying oil and, frying oil [sunflower oil + canola oil]), were considered. Potato and meat of calves (veal) were fried in oil by deep-frying method. All cooking experiments, were conducted by the use of natural gas as the energy source because it is common in residential and commercial settings in Iran. The measurements were conducted in chemistry laboratory of Gonabad University of Medical Sciences, Gonabad, Iran. The sampling campaign was a stove powered by natural gas supplied from gas distribution system and a cooker, and an analyzer for PM. Samples of $PM_{2.5}$ were measured at the height 1.20 m above floor at the height of the respiratory system of a human 30 cm away from the gas stove. Firstly, a pan heated up and then some oil (non-frying oil or, frying oil) was added to the hot pan and let oil heating until ready for frying. Then potato and meat (separately) was transferred to the hot pan. The air during the potato and meat frying was sampled using a set of sorbent tubes and filters. Each test was repeated for three times at three different temperatures (150, 190, 240 °C). Totally, 144 air samples (for two foods, two oils and 3 temperatures) were collected and analyzed for $PM_{2.5}$. During the sampling period, the range of room air temperature was in the range from 21.5 to 23.4 °C. In the current study, all laboratory windows/doors were closed to prevent the infiltration and subsequent influence of ambient air pollutants on the indoor concentrations of $PM_{2.5}$. Mechanical ventilation was not used and hoods and also exhaust fans were in OFF position. The measurement of $PM_{2.5}$ was conducted by a Particulate Air Monitoring Equipment (HAZ-DUST Model EPAM-5000, USA).

### Estimation of human health risk

Health risk associated with the inhalation of $PM_{2.5}$ was estimated using a method developed by the US EPA. In this study, non-cancer risk of $PM_{2.5}$ during potato and meat frying was estimated for adults. For the estimation of non-cancer risk, hazard quotients (HQ) values from inhalation of total $PM_{2.5}$ were calculated. In this study, HQs from exposure to $PM_{2.5}$ were quantified for cooks using following equations [[14], [15], [16], [17]]:

(1) $$\text{EDI}{\;}_{\text{Inhalation}}=\left(\text{CP}M_{2.5}\times \;\text{InhR}\;\times \;\text{EF}\;\times \;\text{ED}\;\times 10^{-3}\right)/\text{BW}\;\times \;\text{AT}$$

or

(2) $$\begin{array}{c} \mathrm{EC}=\left(\text{CP}M_{2.5}\times \;\text{EF}\;\times \;\text{ED}\;\times 10^{-3}\right)/\text{AT} \end{array}$$

(3) HQ=(EDI)/RfDorHQ=(EC)/RfC

where, EDI is the estimated daily intake of PM (mg/kg/day) and EC is exposure concentration in air (mg/m^3^). InhR is the average daily inhalation rate of air (m^3^/day), ED is exposure duration (year/s), EF signifies exposure frequency (days/year), BW is body weight (kg) and AT shows averaging exposure time (day/s), RfD is reference dose (mg/kg/day) and RfC (mg/m^3^) is reference concentration of $PM_{2.5}$, respectively. Table 1 outlines the exposure parameters for the quantification of health risks of $PM_{2.5}$ during potato and meat frying. The data collected in the present study was analyzed using Microsoft Excel 2016.

**Table 1:** Factors utilized for human health risk assessment of $PM_{2.5}$.

Abbreviation	Definition	Refs.		
		Unit	Cooks	
EF	Exposure frequency	days/years	121	[18]
BW	Body weight	kg	80	[17]
ED	Exposure duration	years	30	[17]
InhR	Inhalation rate	m^3^/day	20	[19]
AT	Averaging timing of non-carcinogenic exposure	days	8 hr/24 hr*365= 121.6	[17]
$C_{\text{PM}}$	Concentration of particulate matters	µg/m^3^		
$10^{-3}$	Conversion factor	mg/µg	0.001	
RfC	Inhalation reference concentration of $PM_{2.5}$	mg/kg/day	0.0102	[20]

In general, HQs ≥ 1 suggest the potential non-carcinogenic health risk, i.e., the detrimental health effects on the exposed individuals [21].

### Protocol validation

**Fig. 1:**
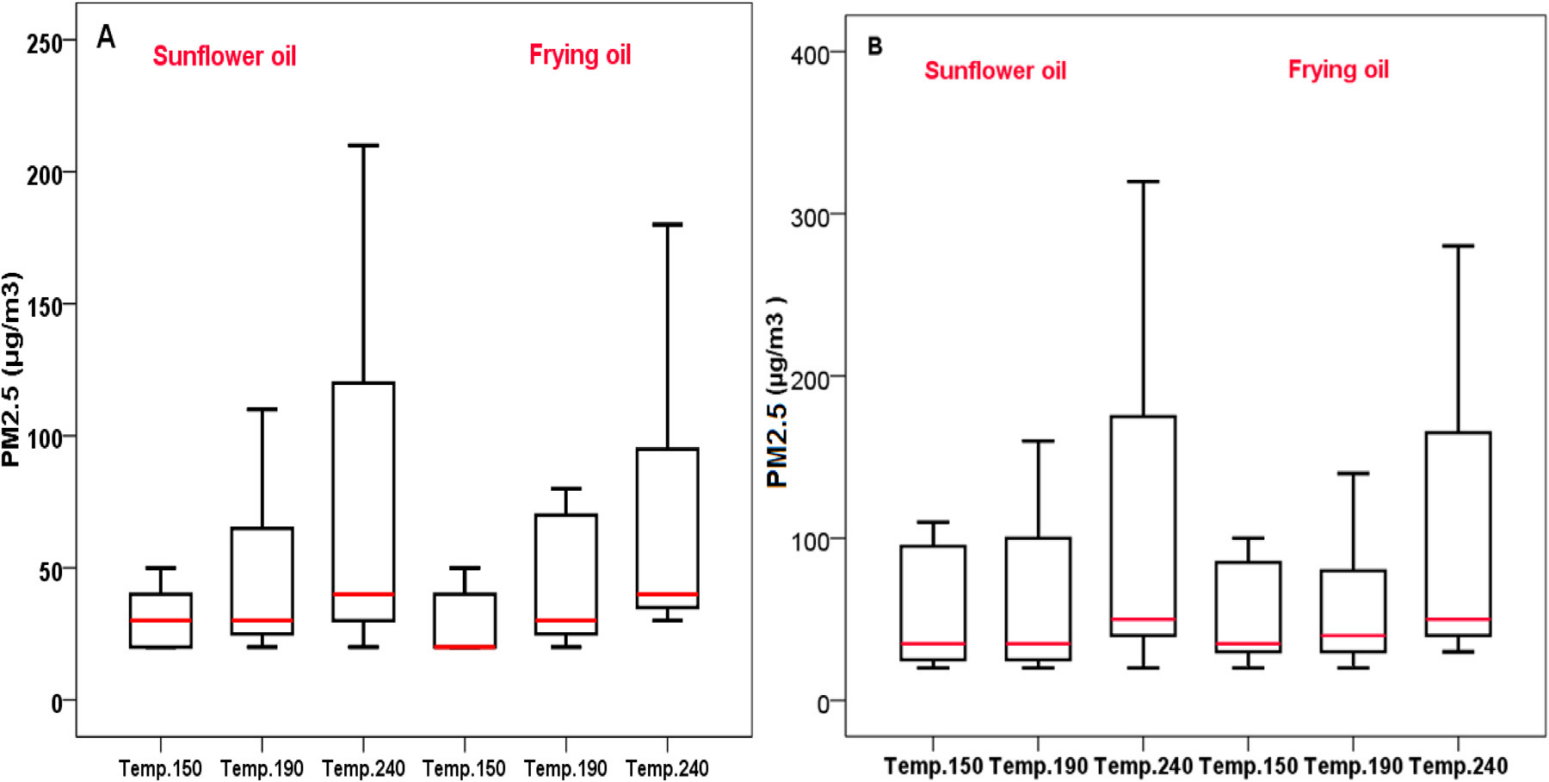
PM2.5 released during (A) potato frying and, (B) meat frying using sunflower and frying oil.

**Fig. 2:**
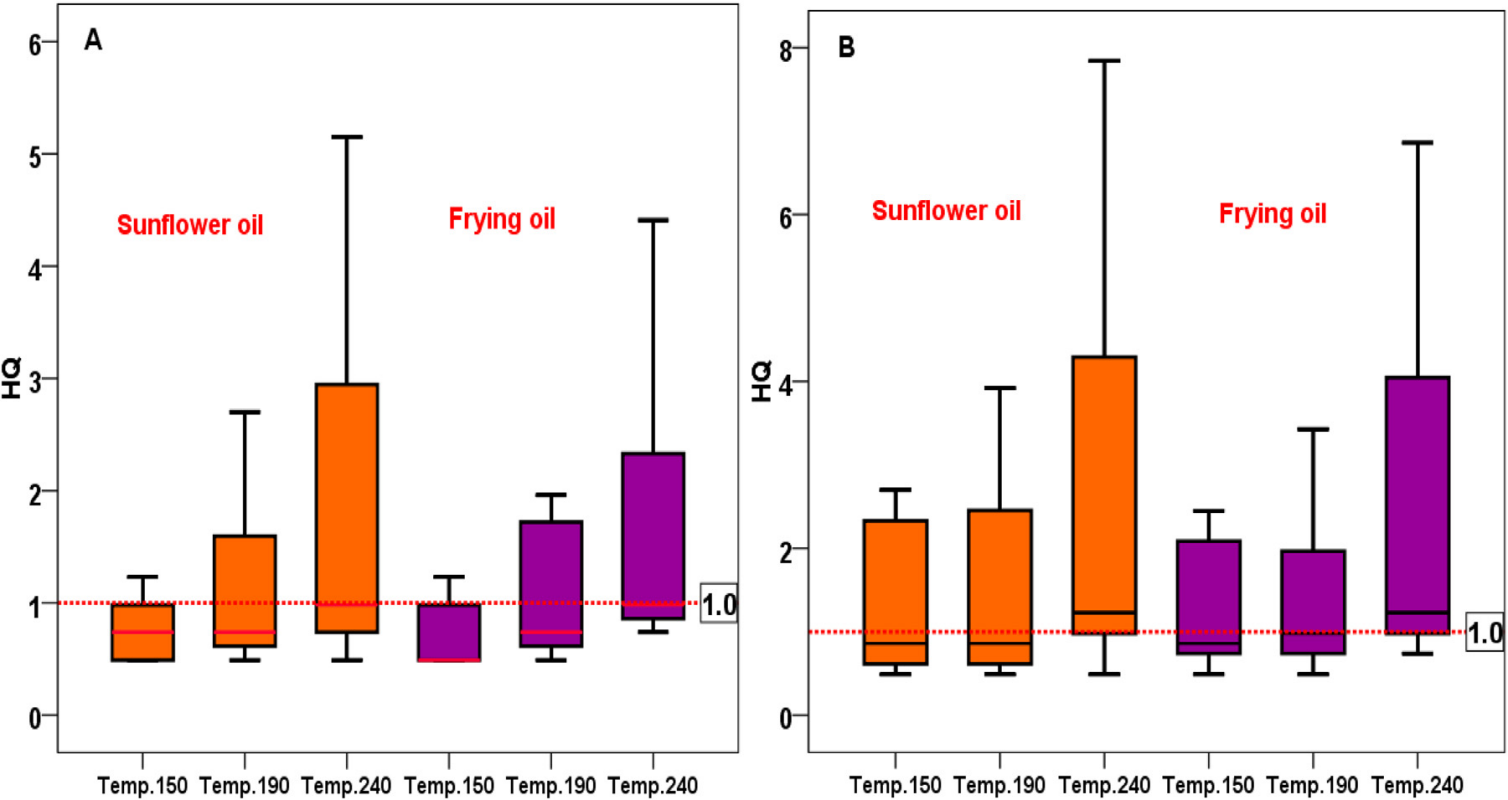
Non-cancer risk via the inhalation of PM_2.5_ released during (A) potato frying and, (B) meat frying using sunflower and frying oil for cooks.

Levels of $PM_{2.5}$ released during potato frying and meat frying using sunflower and frying oil is shown in Fig. 1. As can be seen in the figure, levels of $PM_{2.5}$ increases with temperature increase. Non-frying oil released more $PM_{2.5}$. In total, meat frying released higher levels of $PM_{2.5}$ compared to potato. Table 2, Table 3 summarizes chronic daily intake through inhalation of $PM_{2.5}$ during meat frying using sunflower and frying oil for cooks. Higher levels of $PM_{2.5}$ exposure occurred during meat frying and at higher cooking temperatures. Human health risk via the inhalation of $PM_{2.5}$ released during potato frying and meat frying using sunflower and frying oil for cooks is depicted in Fig. 2. The results indicated health hazards for adults via inhalation of $PM_{2.5}$ during potato and meat frying.

**Table 2:** Estimated daily intake through inhalation of PM_2.5_ during potato frying using sunflower and frying oil for cooks.

Sunflower oil			Frying oil		
Temperature					
150 ^o^ C	190 ^o^ C	240 ^o^ C	150 ^o^ C	190 ^o^ C	240 ^o^ C
0.005	0.005	0.0075	0.005	0.005	0.0075
0.01	0.0225	0.0425	0.01	0.02	0.045
0.005	0.0075	0.01	0.005	0.0075	0.0125
0.005	0.005	0.005	0.005	0.0075	0.0075
0.0125	0.0275	0.0525	0.0125	0.0175	0.04
0.0075	0.01	0.0125	0.0075	0.01	0.01
0.005	0.005	0.0075	0.005	0.005	0.0075
0.01	0.0175	0.0275	0.01	0.0175	0.025
0.005	0.0075	0.0075	0.005	0.005	0.01
0.0075	0.0075	0.0075	0.005	0.0075	0.01
0.0125	0.015	0.0325	0.01	0.02	0.0225
0.0075	0.0075	0.01	0.005	0.0075	0.01

**Table 3:** Estimated daily intake through inhalation of PM_2.5_ during meat frying using sunflower and frying oil for cooks.

Sunflower oil			Frying oil		
Temperature					
150 ^o^ C	190 ^o^ C	240 ^o^ C	150 ^o^ C	190 ^o^ C	240 ^o^ C
0.005	0.005	0.01	0.005	0.005	0.01
0.0275	0.04	0.08	0.025	0.035	0.07
0.0075	0.01	0.015	0.0075	0.01	0.0125
0.005	0.005	0.005	0.0075	0.0075	0.0075
0.025	0.035	0.07	0.02	0.0325	0.06
0.0075	0.0075	0.0125	0.01	0.01	0.0125
0.005	0.005	0.0075	0.005	0.005	0.0075
0.0225	0.0275	0.0475	0.0225	0.0225	0.04
0.0075	0.0075	0.01	0.0075	0.01	0.01
0.01	0.0075	0.01	0.0075	0.0075	0.01
0.025	0.0225	0.04	0.0225	0.0175	0.0425
0.0125	0.01	0.0125	0.01	0.0125	0.0125

## Limitations

This work is pioneering in its focus on potato, meat and oils, which many people consume them on a daily basis, making it an important work. Because of time and budget constraints, the work could not be extended to real environment (kitchens), which may limit the applicability of the results.

## CRediT authorship contribution statement

**Mehdi Qasemi:** Conceptualization, Methodology, Software, Validation, Data curation, Writing – original draft, Visualization, Investigation, Supervision, Software, Validation. **Ali Alami:** Conceptualization, Methodology, Software. **Ahmad Zarei:** Validation, Data curation, Writing – original draft, Visualization, Investigation, Supervision, Software, Validation, Writing – review & editing. **Mahmoud Shams:** Conceptualization, Methodology, Software. **Mojtaba Afsharnia:** Conceptualization, Methodology, Software.

## Declaration of competing interest

The authors declare that they have no known competing financial interests or personal relationships that could have appeared to influence the work reported in this paper.

## Data Availability

Data will be made available on request.
